# Detecting Spatiotemporal Patterns of Newly Diagnosed HIV Infection in the China-Myanmar Border Region, 2010 to 2022: Longitudinal Observational Study

**DOI:** 10.2196/81767

**Published:** 2026-05-20

**Authors:** Jin Yang, Qiyu Zhu, Pei Liu, Jibao Wang, Xing Duan, Yikui Wang, Shijiang Yang, Yuecheng Yang, Runhua Ye, Peng Xu, Song Duan, Cong Jin

**Affiliations:** 1 Dehong Prefecture Center for Disease Control and Prevention, Mangshi, China; 2 National Center for AIDS/STD Control and Prevention, Chinese Center for Disease Control and Prevention, 155 Changbai Road Changping District, Beijing, 102206, China, 86 10 58900922; 3 National Key Laboratory of Intelligent Tracking and Forecasting for Infectious Diseases, National Center for AIDS/STD Control and Prevention, Chinese Center for Disease Control and Prevention, Beijing, China

**Keywords:** HIV, spatial analysis, spatiotemporal heterogeneity, geographic mapping, Bayesian model, public health

## Abstract

**Background:**

Geospatial analysis plays an essential role in informing targeted human immunodeficiency virus (HIV) intervention. The Dai-Jingpo Autonomous Prefecture of Dehong (hereinafter referred to as Dehong), located along the China-Myanmar border in the Yunnan province, has been heavily impacted by HIV infection. Given the complex local epidemic context, particularly frequent cross-border population movement, there is an urgent need to apply spatiotemporal analytical approaches to guiding resource allocation. Existing evidence has demonstrated the substantial spatial variations of newly diagnosed HIV infection this region. However, these spatiotemporal variations have not been fully explored at a finer geographic and temporal resolution.

**Objective:**

This study aims to comprehensively investigate the spatiotemporal variations of newly diagnosed HIV infection at a finer scale in this border region to inform targeted interventions.

**Methods:**

Data on newly diagnosed HIV cases at the township level in Dehong were collected from 2010 to 2022. The rate of newly diagnosis HIV cases was calculated annually. GeoDetector *q* statistics were performed to assess the spatially stratified heterogeneity of the rate of newly diagnosed HIV cases. The Bayesian space-time hierarchical model was applied to detect the spatiotemporal patterns of newly diagnosed HIV infection across the region.

**Results:**

A total of 5045 newly diagnosed HIV cases were identified in Dehong from 2010 to 2022. The rate of newly diagnosed HIV cases decreased from 57.1 cases per 100,000 population in 2010 to 13.3 cases per 100,000 population in 2022, a decrease of 76.7% over the past 13 years. The overall temporal relative risk decreased from 2.11 (95% CI 1.84‐2.41) in 2010 to 0.48 (95% CI 0.40‐0.56) in 2022. There was substantial spatiotemporal heterogeneity in the risk of newly diagnosed HIV infection, with townships near the China-Myanmar border having a higher spatial relative risk. Notable spatially stratified heterogeneity in the rate of newly diagnosed HIV cases when stratified by the distance of townships to the China-Myanmar border was observed (*q*=0.27; *P*=.004). Among the 51 townships in Dehong, 22 (43.1%) hotspots and 22 (43.1%) coldspots were identified. Notably, in comparison to the overall declining temporal trend, 2 hotspots and 4 coldspots exhibited a slower declining trend, suggesting that these regions may require additional intervention efforts.

**Conclusions:**

This study comprehensively estimated the spatiotemporal risk of newly diagnosed HIV infection across Dehong, revealing high-risk areas concentrated near the China-Myanmar border. Priority should be given to implementing targeted interventions to control cross-border HIV transmission, including the establishment of cross-border HIV control mechanisms, as well as the strengthening of management measures for cross-border populations. Furthermore, this study offers methodological insights into the use of routine surveillance data and Bayesian spatiotemporal modeling to better understand HIV transmission dynamics at finer geographic scales and to support precision-oriented HIV prevention services.

## Introduction

Since the acquired immune deficiency syndrome (AIDS) cases were first reported in 1981, there are now 40.8 million people living with human immunodeficiency virus (HIV) globally [1]. To prevent the transmission of HIV, the Joint United Nations Programme on HIV/AIDS (UNAIDS) has proposed the 95-95-95 targets, which aim to end HIV/AIDS as a public health threat by 2030 [2]. In China, impressive progress has been made to reduce new HIV infection and enhance the health quality of people living with HIV. The steadily increasing of antiretroviral therapy (ART) coverage in China has led to a significantly reduction in all-cause mortality, from 5.4% in 2013 to 2.7% in 2022 [3]. Achieving the goal of ending HIV/AIDS as a public health threat will increasingly depend on the implementation of precise and context-specific intervention strategies tailored to local epidemic characteristics.

The Dai-Jingpo Autonomous Prefecture of Dehong (hereinafter referred to as Dehong) in the Yunnan province, located in the China-Myanmar border, is an area severely affected by HIV infection, where the first epidemic outbreak of HIV in China was recognized [4]. Located on major illicit drug-trafficking routes from the “Golden Triangle,” a primary opium-producing area in Southeast Asia, early HIV transmission in this region was predominantly driven by injecting drug users (IDUs) [5]. After years of fighting illicit drugs and providing HIV harm reduction services to IDUs, such as syringe exchange programs and methadone maintenance treatment, sexual contact has become the dominant route of HIV transmission [6]. Consequently, the local HIV epidemic has become increasingly complex and concealed.

To achieve effective control of the epidemic, it is essential to accurately understand the current trends in HIV infection. Spatial analysis is often used to reflect the severity of the HIV epidemic at finer geographic scales and to inform the allocation of health resources and targeted interventions [7]. A previous study conducted in Dehong analyzed the spatial patterns of different years using HIV case data from 1989 to 2018 [8]. However, this previous study only performed purely spatial analyses based on annual newly diagnosed data and did not incorporate temporal terms. As local governments dynamically adjust policies and measures in response to evolving epidemic conditions, the risk and trends of HIV infection may vary over time across different regions of Dehong. Traditional spatial models, which only capture geographic variation, fail to integrate temporal dynamics of HIV transmission.

Understanding fine-scale spatiotemporal heterogeneity has become an increasingly important methodological challenge in HIV epidemiology, particularly in high-burden or highly heterogeneous settings. In this study, we employed a Bayesian space-time hierarchical model (BSTHM) to retrospectively analyze data of newly diagnosed HIV cases in Dehong from 2010 to 2022, enabling us to detect the spatiotemporal heterogeneity of HIV transmission. Compared to classical spatial analysis models, which are used to examine spatial autocorrelation and depict the spatial distribution of diseases, BSTHM is suitable for analyzing spatial data with continuous temporal variation. BSTHM effectively integrates spatiotemporal correlations and decomposes spatiotemporal data into 3 key components: common spatial patterns, common temporal patterns, and potential spatiotemporal interactions [9]. Therefore, BSTHM can be used to analyze the spatiotemporal heterogeneity and interactions of newly diagnosed HIV cases in Dehong from 2010 to 2022, providing more comprehensive and detailed data to reveal the trends of the HIV epidemic at both global and local levels over time.

This study aims to systematically describes the spatiotemporal distribution and changing characteristics of newly diagnosed HIV cases in Dehong. By applying a Bayesian spatiotemporal hierarchical modeling approach to routine HIV surveillance data, this study explores the potential of maximizing the utility of existing monitoring systems to characterize epidemic patterns at finer spatial and temporal scales. The findings will enhance the understanding of local epidemic characteristics, thereby guiding targeted interventions and resource allocation, further curbing the spread of the epidemic and effectively reducing new HIV infections in the area. In addition, this analytical approach may be applicable to understanding localized HIV transmission patterns and informing targeted intervention strategies in other regions.

## Methods

### Study Area

**Figure 1.**
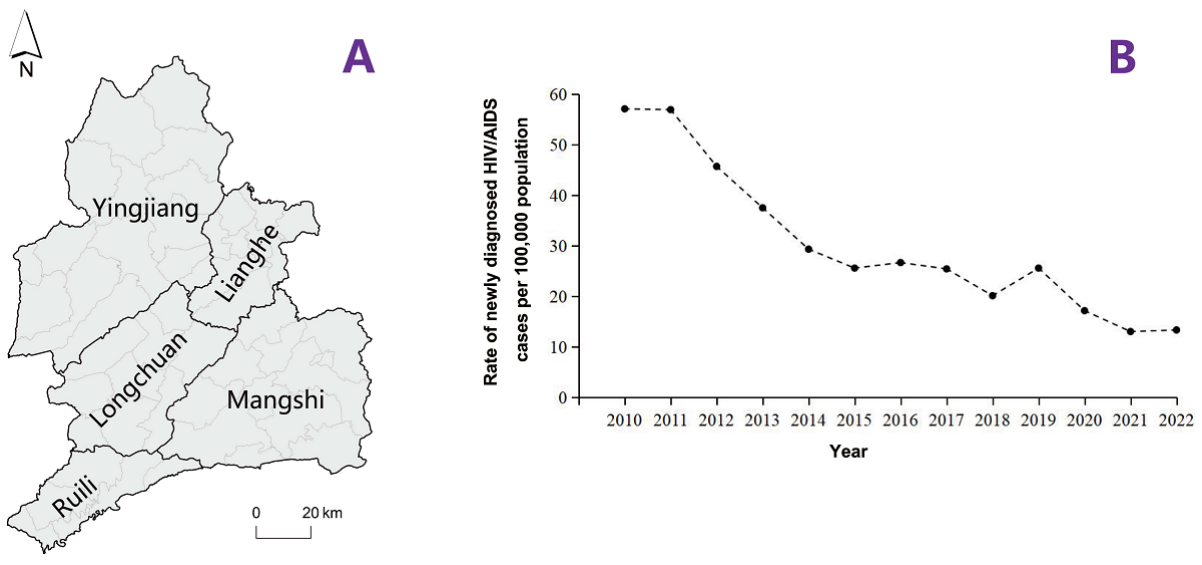
Administrative map and rate of newly diagnosed HIV/AIDS cases in the Dai-Jingpo Autonomous Prefecture of Dehong in the Yunnan province. (A) Administrative map. (B) The rate of newly diagnosed HIV/AIDS cases per 100,000 population from 2010 to 2022. AIDS: acquired immune deficiency syndrome; HIV: human immunodeficiency virus.

This longitudinal observational study was conducted in Dehong (Figure 1A), which has a population of approximately 1.3 million and an area of 11,526 km^2^. The study region is divided into 5 districts (Ruili, Mangshi, Lianghe, Yingjiang, and Longchuan), encompassing 51 township-level districts (6 in Ruili, 12 in Mangshi, 9 in Lianghe, 15 in Yingjiang, and 9 in Longchuan).

### Data Sources

Data on newly diagnosed HIV cases at the township level in Dehong were obtained from the national HIV/AIDS Comprehensive Response Information Management System (CRIMS) by the Dehong Center for Disease Control and Prevention. The data cover the period from January 1, 2010, to December 31, 2022, and include HIV cases that met the following criteria: (1) reported in Dehong; (2) resided in the specific townships within Dehong; and (3) of Chinese nationality. Township-level population data were obtained from the 2020 China Population Census [10]. All data were collected at the township level. The distance from each township to the China-Myanmar border was defined as the shortest distance between the township administrative boundary and the international border. Based on the calculated distances, all townships were classified into 4 distance-based groups according to proximity, which served as the stratification variable in the GeoDetector analysis. The rate of newly diagnosis HIV cases was obtained by dividing the number of newly diagnosed cases by the local resident population.

### GeoDetector *q* Statistics

As reported in previous studies, the HIV transmission in Dehong may be associated with cross-border population movements between China and Myanmar. We investigated the spatially stratified heterogeneity (SSH) of newly diagnosed HIV case rates, stratified by the distance of the township to the China-Myanmar border. The GeoDetector *q* statistics were calculated to measure the SSH of newly diagnosed HIV case rates across different township-level units in Dehong, and the formula is as follows [11]:


(1) $$q=1-\frac{1}{N\sigma^{2}}\sum_{h=1}^{L}N_{h}\sigma_{h}^{2}$$

In the formula (Eq. 1), N is the total number of townships in Dehong Prefecture, $\sigma^{2}$ denotes the variance of newly diagnosed HIV case rates across all townships, $N_{h}$ refers the number of townships in stratum h (1, 2, …, L), and $\sigma_{h}^{2}$ represents the variance of newly diagnosed HIV case rates within the stratum h. The *q* value ranges from 0 to 1, with a value of 0 indicating complete randomness and 1 representing perfect stratified heterogeneity.

### BSTHM Formulas

The Poisson and log link regression functions used to perform analyses were as follows:


(2) $$y_{it}∼Poisson(n_{i}r_{it})$$

(3) $$\log(r_{it})=\alpha +S_{i}+U_{i}+(b_{0}t^{*}+v_{t})+b_{1i}t^{*}+\varepsilon_{it}$$

In the Poisson likelihood function (Eq. 2), $y_{it}$ represents the number of newly diagnosed HIV cases in township i at year t, $n_{i}$ denotes the total population in township i, and $r_{it}$ is the modeled risk of newly diagnosed HIV/AIDS cases in township i at year t.

In the log link regression function (Eq. 3) that describes the risk of newly diagnosed HIV infection, α is the overall log disease risk for the entire study period, and ($S_{i}+U_{i}$) is the disease risk in township i. Specifically, $S_{i}$ captures the spatially structured component, and $U_{i}$ represents the spatially unstructured component. The term $t^{*}$ denotes the median time of the entire study period, with $\left(b_{0}t^{*}+v_{t}\right)$ and $b_{1i}t^{*}$ specifying the overall and local temporal trend in township i, respectively. Specifically, the term $b_{0}t^{*}$ captures the linear trend, while $v_{t}$ accounts for the nonlinearity in the overall trend pattern. Given the observed decrease in the newly diagnosed HIV cases rate of over the study period, if $b_{1i}$ < 0, it indicates that the decreasing trend in township i is faster than the overall decreasing trend. Conversely, $b_{1i}$ > 0 suggests a slower trend relative to the overall decrease. The Gaussian noise was captured by $\varepsilon_{it}$.

### Area Classification by BSTHM

The study area was classified into 9 categories (3 risk categories × 3 trend categories) based on the posterior probability of *P* (exp ($S_{i}$) >1 |data) and *P* ($b_{1i}$ >0 |$h_{i}$, data), following a specific 2-stage criteria [12]. In the first stage, townships were classified into 3 categories: hotspot, coldspot, and not cold/hot spot. A township is classified as a hotspot ($h_{i}$=1) if *P* (exp ($S_{i}$) >1 |data) is greater than 0.8, and as a coldspot ($h_{i}$=2) if this probability is less than 0.2 [9]. The remaining townships were classified as not cold/hot spots ($h_{i}$=3). In the second stage, temporal changes were categorized into 3 classes (faster decrease, common decrease, and slower decrease) according to *P* ($b_{1i}$ >0 |$h_{i}$, data). A township was defined as exhibiting a slower decreasing trend compared to the overall decreasing trend if *P* ($b_{1i}$ >0 |$h_{i}$, data) >0.8, a faster decreasing trend if *P* ($b_{1i}$ >0 |$h_{i}$, data) <0.2, and a common decreasing trend if 0.2 <*P* ($b_{1i}$ >0 |$h_{i}$, data) <0.8 [9]. Consequently, a total of 9 categories were established.

Hyperparameters for prior distributions were chosen following the approach of Li et al [9], which has been widely applied in BSTHM. Convergence of the Markov Chain Monte Carlo chains was evaluated using the Gelman-Rubin statistic, with values below 1.05 indicating satisfactory convergence. The variance partition coefficient was measured to assess model performance. All BSTHM analysis were conducted using WinBUGS 1.4.3.

### Ethical Considerations

This study was approved by the Institutional Review Board of the National Center for AIDS/STD Prevention and Control, Chinese Center for Disease Control and Prevention (approval number: X241028828). The data used in this study were from the CRIMS. No personally identifiable information was present in the data analyzed for this study.

## Results

### Characteristics of Newly Diagnosed HIV Cases

Between 2010 and 2022, a total of 5045 newly diagnosed HIV cases were reported in Dehong. Of these cases, 3235 (64.1%) were male, 2570 (50.9%) were aged between 31 and 49 years at the time of diagnosis, 2120 (42.0%) were of Han ethnicity, and 2937 (58.2%) had a primary school education or less (Table 1). More than half of the cases (n=2713, 53.8%) were married or cohabiting with a partner. In terms of the transmission routes, 4211 (83.5%) were infected through heterosexual contact, followed by 562 (11.1%) through injecting drug use, and 164 (3.3%) through homosexual contact.

**Table 1.** Characteristics of newly diagnosed human immunodeficiency virus (HIV) cases in the Dai-Jingpo Autonomous Prefecture of Dehong in the Yunnan province, 2010‐2022 (N=5045).

Characteristic	Cases, n (%)
Sex	
Male	3235 (64.1)
Female	1810 (35.9)
Age at HIV diagnosis (years)	
≤30	1536 (30.5)
31–49	2570 (50.9)
≥50	939 (18.6)
Ethnicity	
Han	2120 (42.0)
Dai	1509 (29.9)
Jingpo	1079 (21.4)
Others	337 (6.7)
Education	
Primary school or illiterate	2937 (58.2)
Middle school/high school/vocational school	1858 (36.9)
College or above	249 (4.9)
Unknown	1 (0.0)
Marital status	
Single	1183 (23.4)
Married or living with partner	2713 (53.8)
Divorced or widowed	1149 (22.8)
HIV transmission route	
Heterosexual contact	4211 (83.5)
Homosexual contact	164 (3.3)
Injecting drugs	562 (11.1)
Others	108 (2.1)

### Dynamics of Newly Diagnosed HIV Infections

**Figure 2.**
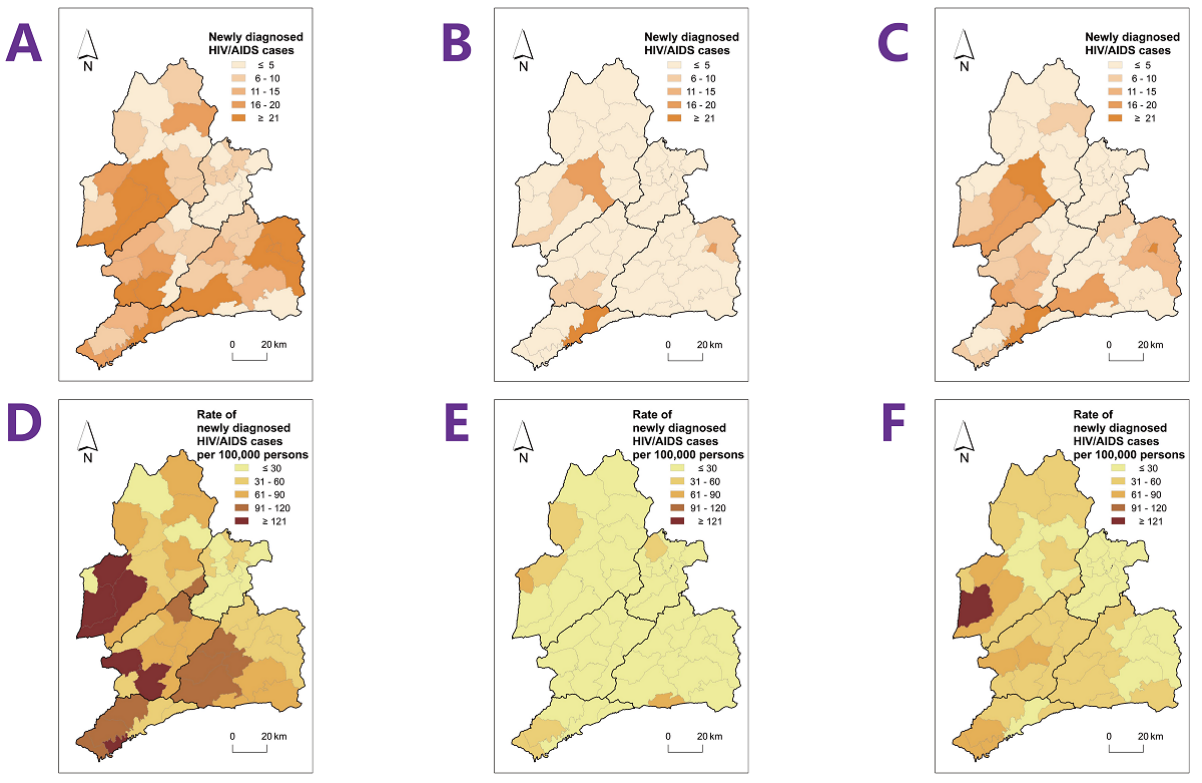
The number and rate of newly diagnosed HIV/AIDS cases in the Dai-Jingpo Autonomous Prefecture of Dehong in the Yunnan province, 2010‐2022. (A) Number of newly diagnosed HIV/AIDS cases in 2010. (B) Number of newly diagnosed HIV/AIDS cases in 2022. (C) Annual average number of newly diagnosed HIV/AIDS cases during 2010‐2022. The color intensity represents the number of newly diagnosed HIV/AIDS cases, with darker colors indicating higher values. (D) Rate of newly diagnosed HIV/AIDS cases per 100,000 population in 2010. (E) Rate of newly diagnosed HIV/AIDS cases per 100,000 population in 2022. (F) Annual average rate of newly diagnosed HIV/AIDS cases per 100,000 population during 2010‐2022. The color intensity represents the rate of newly diagnosed HIV/AIDS cases per 100,000 population, with darker colors indicating higher rates. AIDS: acquired immune deficiency syndrome; HIV: human immunodeficiency virus.

The rate of newly diagnosed HIV cases in Dehong decreased over time, from 57.1 cases per 100,000 population to 13.3 cases per 100,000 population, representing a 76.7% reduction over the past 13 years (Figure 1B). Furthermore, the proportion of townships with a rate of newly diagnosed HIV cases exceeding 60 cases per 100,000 population decreased from 52.9% (27/51) in 2010 to 3.9% (2/51) in 2022. Among the 51 townships in Dehong, the number of townships with more than 20 newly diagnosed cases decreased from 10 in 2010 to 1 in 2022 (Figure 2). GeoDetector analysis revealed notable SSH in the rate of newly diagnosed HIV case when stratified by the distance of townships to the China-Myanmar border (*q*=0.27; *P*=.004).

### Bayesian Analysis of Newly Diagnosed HIV Infections

#### Overall Temporal Trend

**Figure 3.**
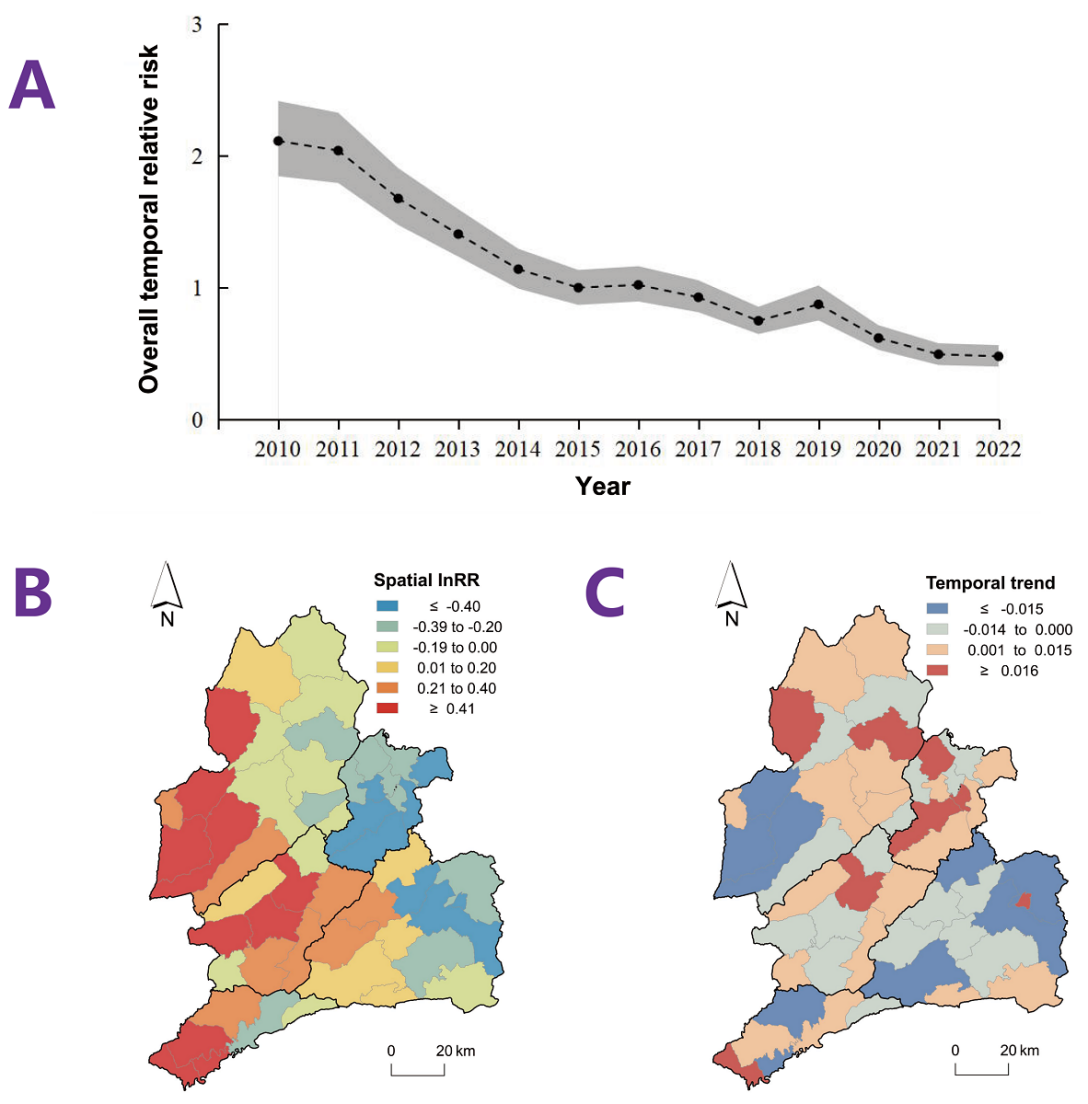
The characteristics of spatiotemporal relative risk of newly diagnosed HIV/AIDS cases in the Dai-Jingpo Autonomous Prefecture of Dehong in the Yunnan province, 2010‐2022. (A) Overall temporal relative risk. The dots represent the estimated overall temporal relative risk [exp($b_{0}t^{*}+v_{t}$)], with the corresponding 95% credible intervals shown as the shaded area. (B) Spatial relative risk. The color intensity represents the estimated spatial relative risk ($S_{i}+U_{i}$) without exponential transformation, where higher values (warmer colors) indicate a relatively higher risk in township i compared to the overall average risk in the study region. (C) Local temporal trend departure from the overall trend. The color intensity represents the estimated local departure trend ($b_{1i}$) without exponential transformation, where the estimated lower value (cooler colors) indicates a faster declining trend compared to the overall declining trend. AIDS: acquired immune deficiency syndrome; HIV: human immunodeficiency virus; InRR: natural logarithm of the spatial relative risk.

The variance partition coefficient for BSTHM was 91.3% (95% CI 85.8%‐95.5%). The BSTHM results indicate a declining trend in newly diagnosed HIV infection over the past 13 years, with the overall temporal relative risk decreasing from 2.11 (95% CI 1.84‐2.41) in 2010 to 0.48 (95% CI 0.40‐0.56) in 2022 (Figure 3A).

#### Geographical Distribution

Substantial geographical variations in the spatial relative risk of newly diagnosed HIV infection were observed across the study area (Figure 3B). Generally, the western region of Dehong experienced relatively higher risks. The estimated local slopes, which measure the departure of local trends from the overall declining trend, revealed that townships depicted in warm color (red and orange) exhibited slower declining trends compared to the overall trend (Figure 3C). Specifically, townships with slower local declining trends than the prefecture-wide level were predominantly located in Yingjiang, Longchuan, and Lianghe.

#### Area Classification

**Figure 4.**
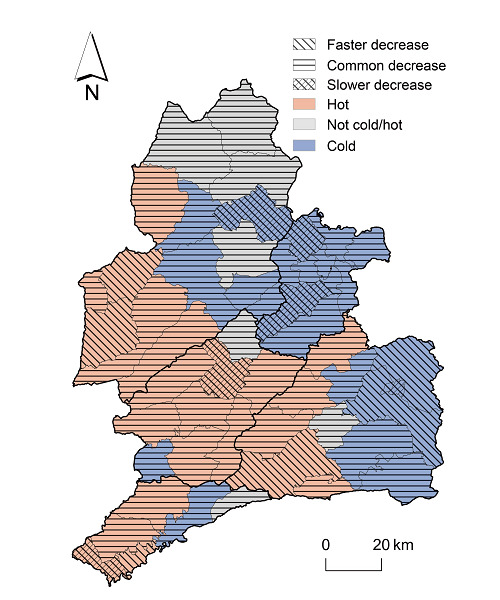
Spatial distribution of hotspots and coldspots for newly diagnosed HIV/AIDS cases in the Dai-Jingpo Autonomous Prefecture of Dehong in the Yunnan province, 2010‐2022. AIDS: acquired immune deficiency syndrome; HIV: human immunodeficiency virus.

Among the 51 townships, 22 (43.1%) were identified as hotspots, 22 (43.1%) as cold spots, and 7 (13.7%) as neither hot nor cold spots (Table 2, MultimediaAppendices 1  2). As shown in Figure 4, the 22 hotspots were mainly distributed in the southwestern part of Dehong Prefecture, near the China-Myanmar border. Among these hotspots, 1 township in Ruili and 1 in Longchuan exhibited a slower declining trend compared to the overall trend. Conversely, 2 hotspots in Yingjiang, 1 in Mangshi, and 1 in Ruili showed a faster declining trend than the overall trend. Among the 22 cold spots, 2 townships in Lianghe, 1 in Mangshi, and 1 in Yingjiang showed a slower declining trend than the overall trend. Meanwhile, 2 cold spots in Mangshi exhibited a faster declining trend than the overall trend. There were 7 townships that were neither hot nor cold spots.

**Table 2.** Area classification based on the Bayesian space-time hierarchy model in the Dai-Jingpo Autonomous Prefecture of Dehong in the Yunnan province, 2010‐2022.

Area classification	Faster decrease	Slower decrease	Common decrease	Total, n (%)
Hotspots	4	2	16	22 (43.1)
Coldspots	2	4	16	22 (43.1)
Not hotspots/coldspots	0	0	7	7 (13.7)

## Discussion

### Principal Findings

In this study, using township-level data on newly diagnosed HIV infection in Dehong from 2010 to 2022, we quantitatively characterized fine-scale spatiotemporal patterns of HIV risk using BSTHM. The number of newly diagnosed HIV cases has declined significantly over the past 13 years, accompanied by notable SSH. Substantial spatiotemporal heterogeneity was observed in the risk of newly diagnosed HIV infection. By jointly modeling spatial structure and temporal dynamics, several townships were identified as areas that are likely to become or remain hotspots in the future.

In our analysis of newly diagnosed HIV infection from 2015 to 2022, IDUs accounted for 11.1% of cases, while heterosexual contact primarily dominated HIV transmission, responsible for 83.5% of cases. Since HIV was first identified among IDUs in Dehong in 1989 [4], the Chinese government has allocated massive resources to curb HIV transmission. At the provincial level, the Yunnan government has initiated multiple campaigns against HIV and illicit drugs, with remarkable achievements. A study incorporating HIV incidence assays to estimate the HIV incidence among IDUs in Dehong estimated that the HIV incidence among Chinese IDUs in Dehong decreased from 3.11% (95% CI 1.59%‐4.64%) in the 2009‐2010 period to 0.58% (95% CI –0.06% to 1.04%) in the 2015‐2017 period [13]. Furthermore, the proportion of IDUs among newly diagnosed male HIV cases in Dehong also showed a downward trend, decreasing from 46.7% in 2012 to 32.0% in 2016 [6]. These shifts reflect a structural transition in transmission dynamics, with heterosexual contact becoming the leading cause of local HIV infections, accounting for 84.7% of newly diagnosed HIV cases during the 2015‐2017 period [14]. In 2017, the Yunnan government issued an implementation plan aimed at achieving the 90-90-90 HIV targets by the end of 2020. These unprecedented efforts to strengthen HIV prevention, testing, and treatment have resulted in a significant reduction of 76.7% in the rate of newly diagnosed HIV cases over the past 13 years.

Notable SSH was observed in newly diagnosed HIV cases rate across Dehong (*q*=0.27; *P*=.004). The spatial relative risk was apparently higher in townships closer to the China-Myanmar border and declined with increasing distance (Figure 3B). Consistent with the area classification analysis, most hotspots were concentrated in the cross-border region. Cross-border mobility and high HIV prevalence of Burmese individuals may play a significant role in HIV transmission. Due to lower income levels in Myanmar, many Burmese individuals migrate to China in pursuit of better economic opportunities, resulting in frequent interactions between the 2 populations. Previous studies have noticed the high HIV prevalence among Chinese-Burmese mixed couples [15,16]. Chen et al [17] recruited 617 Yunnan-Myanmar IDUs in 3 counties of Yunnan and conducted molecular transmission analysis on 117 (19.0%) people living with HIV. Their findings indicate that Yunnan-Myanmar IDUs play a pivotal role in the bidirectional cross-border HIV transmission [17]. Hu et al [18] further demonstrated the strong association between Burmese immigrants and HIV transmission in Dehong. According to a survey of 393 Burmese immigrants in Dehong in 2020, they had low levels of HIV knowledge, condom usage, and willingness to undergo HIV testing [19]. Consequently, the convergence of high background prevalence, frequent population mobility, and constrained access to HIV services likely contributes to the persistent elevated risk observed in border townships. Furthermore, in districts such as Ruili and Longchuan, where ports are in close proximity to main residential and commercial areas, the frequency of cross-border mobility and interactions is increased.

To control the cross-border transmission of HIV, several measures have been implemented in Dehong. These include strengthening comprehensive management of cross-border populations, providing free ART to Chinese-Burmese mixed couples or long-term resident immigrants living with HIV, and expanding HIV testing among immigrants engaged in long-term employment in Dehong. Nevertheless, the persistence of high-risk hotspots identified through BSTHM suggests that additional, more precisely targeted interventions are needed. The identification of these townships provides important epidemiological clues for prioritizing resource allocation. Further prevention and control measures are required for hotspot areas of cross-border HIV transmission. First, cross-border HIV prevention and control mechanisms should be established by strengthening bilateral collaborations between China and Myanmar. Second, programs should be implemented to improve health education, promote condom use, and increase HIV testing rates among Burmese immigrants. Third, appropriate HIV treatment programs should be explored for short-term Burmese immigrants, as they are currently ineligible for China’s free ART program. Lastly, efforts should be made to explore ways to improve treatment adherence among HIV-infected immigrants to prevent treatment failure and the development of drug resistance.

In this study, we further estimated the local temporal trend of the spatial pattern using BSTHM, which can predict the spatial pattern shift in the future. The local trends were estimated by comparing their trend patterns with the overall trend pattern. In this study, we found that 2 hotspots exhibited a slower declining trend than the overall trend, while 4 coldspots with a slower declining trend showed a tendency to become “hot” (Table 2). These townships exhibited higher inclination of hotspots in the future and should be prioritized for targeted interventions. Further in-depth epidemiological investigations are warranted to explore the local drivers of transmission in these areas and to inform more precise and context-specific intervention strategies. To our knowledge, few studies in China have investigated the combined spatial patterns and temporal trends in the rate of newly diagnosed HIV infections. Previous spatial analysis studies primarily employed spatial autocorrelation analysis methods to examine the spatial distribution characteristics separately across different time periods [20-24] and used spatial scan statistics to detect cluster areas [21,25]. None of these studies incorporated spatiotemporal correlations. This study modeled spatial relative risk, overall temporal trends, and local temporal trends at the township level, thereby constructing a multifaceted picture of the evolving risk of newly diagnosed HIV cases across Dehong, representing a methodological advantage over traditional spatial analyses. This small area analysis has important implications for the allocation of resources in controlling HIV transmission in similar settings, as interventions and measures can be directly implemented in hotspots or potential hotspots to reduce new infections.

This study is subject to several limitations. First, since HIV diagnoses are not made immediately after HIV infection, the incidence rate could not be calculated in this study. Nevertheless, in the context of generally increasing HIV testing coverage in Dehong, the observed decline in newly diagnosed cases likely reflects, at least to some extent, a reduction in new HIV infections, indicating the effectiveness of local HIV control measures. Second, the analysis in this study was conducted at the township level; the macro-level factors potentially associated with the rate of newly diagnosed HIV infections were not investigated due to the data availability. Third, all analyses in this study were restricted to newly diagnosed HIV cases of Chinese nationality, as data on foreign nationals were not available. Consequently, the findings primarily reflect HIV infection patterns among the local resident population and may underestimate the overall contribution of cross-border populations to the regional HIV epidemic.

### Conclusions

In conclusion, the rate of newly diagnosed HIV cases in Dehong has significantly decreased from 2010 to 2022. By applying a BSTHM framework to routine surveillance panel data, this study reveals substantial variations in the spatial distribution and temporal trends of the risk of newly diagnosed HIV infections within Dehong, with high-risk areas concentrated in townships near the China-Myanmar border. Future efforts should focus on exploring effective measures to reduce new infections in these high-risk areas. This analytical approach provides a new perspective for assessing local needs, guiding resource allocation, and developing targeted interventions to control HIV infection in other high-burden or highly heterogeneous settings.

## Supplementary material

10.2196/81767 Multimedia Appendix 1 Posterior probability of spatial relative risk.

10.2196/81767 Multimedia Appendix 2 Posterior probability of temporal trend.

## Abbreviations

AIDS: acquired immune deficiency syndrome
ART: antiretroviral therapy
BSTHM: Bayesian space-time hierarchical model
CRIMS: HIV/AIDS Comprehensive Response Information Management System
HIV: human immunodeficiency virus
IDU: injecting drug user
SSH: spatially stratified heterogeneity
UNAIDS: Joint United Nations Programme on HIV/AIDS
