# Supplementary figures and images for: Detecting Spatiotemporal Patterns of Newly Diagnosed HIV Infection in the China-Myanmar Border Region, 2010 to 2022: Longitudinal Observational Study
Source: JMIR Public Health Surveill. 2026 May 20;12:e81767. doi: 10.2196/81767 (PMC13189572; doi:10.2196/81767)

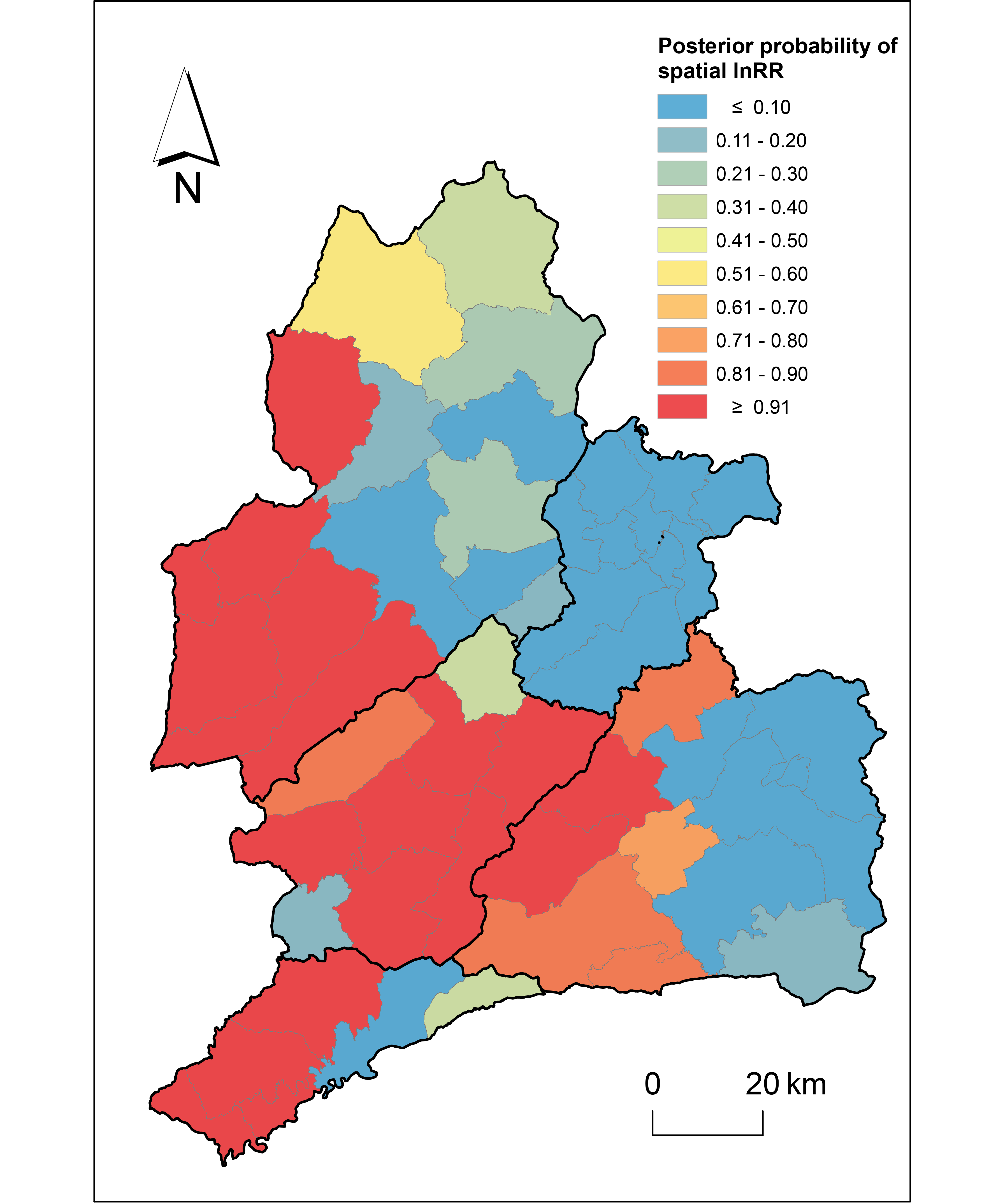

Supplement: Multimedia Appendix 1 [file publichealth-v12-e81767-s001.png]

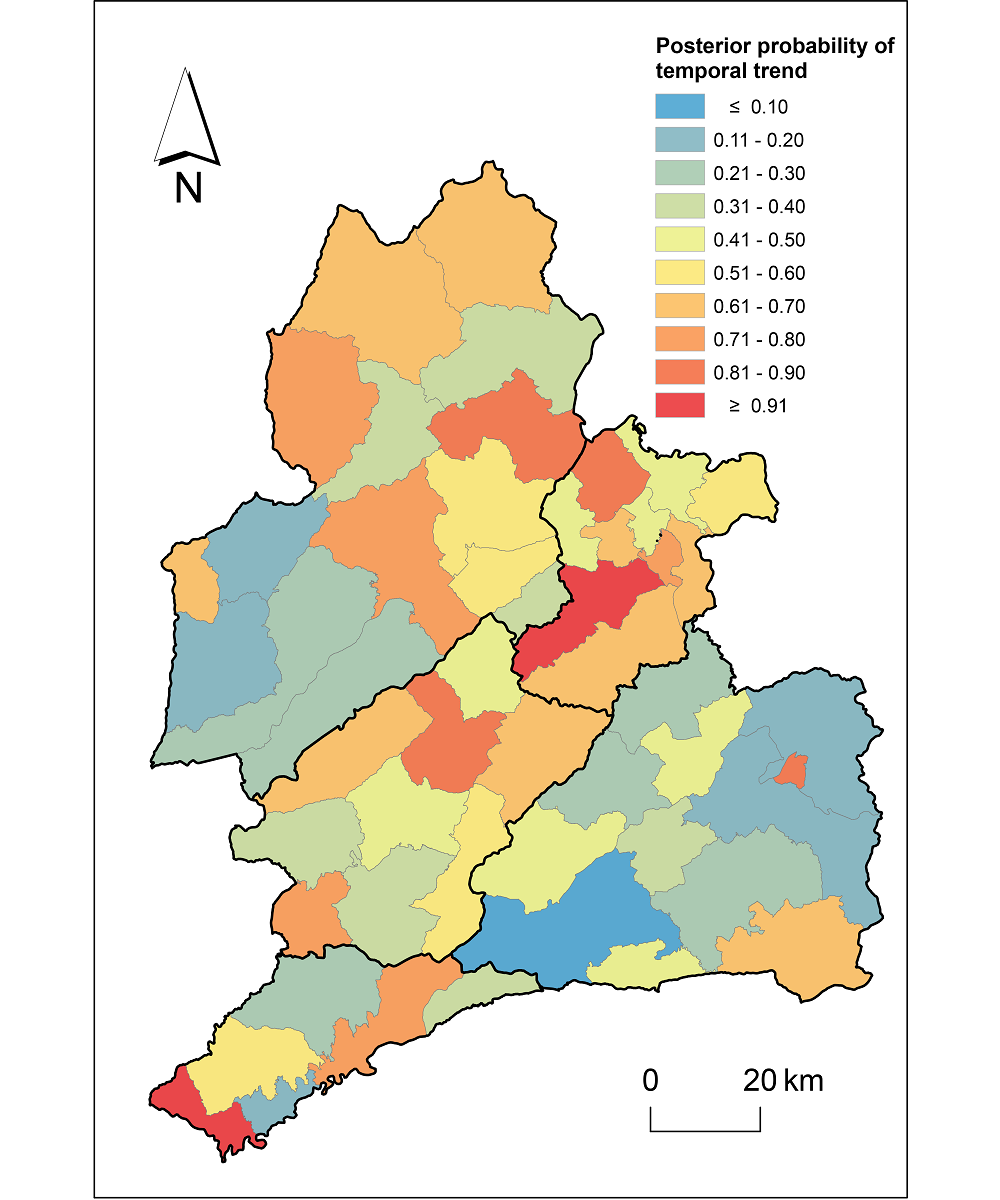

Supplement: Multimedia Appendix 2 [file publichealth-v12-e81767-s002.png]
